# Loneliness and Profiles of Objective and Subjective Memory During Midlife

**DOI:** 10.1093/geroni/igaa057.1868

**Published:** 2020-12-16

**Authors:** Karra Harrington, Nelson Roque, Ruixue Zhaoyang, Jacqueline Mogle, Martin Sliwinski

**Affiliations:** 1 Pennsylvania State University, University Park, Pennsylvania, United States; 2 The Pennsylvania State University, University Park, Pennsylvania, United States; 3 Penn State University, University Park, Pennsylvania, United States

## Abstract

Loneliness is a risk factor for dementia, however it’s relationship with cognitive health during midlife is unclear. We evaluated whether loneliness was associated with profiles of objective and subjective memory in younger and middle-aged adults. Participants (aged 25 to 64 years) underwent an initial loneliness assessment, followed by 14-days of momentary (5 per day) cognitive assessments (objective memory) and daily ratings of memory (subjective memory). Cluster analysis was conducted using person-level means of objective and subjective memory. Three clusters were identified: (1) highest objective and subjective memory (9%); (2) lowest subjective but not objective memory (84%); (3) lowest objective but not subjective memory (7%).There was a trend for higher levels of loneliness in Cluster 2 relative to Clusters 1 and 3. Results suggest that loneliness is more closely related with subjective than objective memory during midlife and are informative for development of interventions targeting cognitive health. Part of a symposium sponsored by the Measurement, Statistics, and Research Design Interest Group.

